# Early Infant Feeding of Formula or Solid Foods and Risk of Childhood Overweight or Obesity in a Socioeconomically Disadvantaged Region of Australia: A Longitudinal Cohort Analysis

**DOI:** 10.3390/ijerph15081685

**Published:** 2018-08-07

**Authors:** Haider Mannan

**Affiliations:** Translational Health Research Institute, Western Sydney University, Campbelltown, NSW 2560, Australia; h.mannan@westernsydney.edu.au; Tel.: +61-02-4620-3951

**Keywords:** childhood obesity, infant feeding factors, southwestern Sydney

## Abstract

In southwestern Sydney the timing of introduction of formula and solids may be associated with risk of childhood overweight or obesity, and this may vary by age at breastfeeding cessation during first year. We included 346 infants from southwestern Sydney using the longitudinal study for Australian children (LSAC), who at baseline were singleton, full term, and normal weight births. The outcome risk of overweight or obesity was measured at every two-year interval of children aged 0 or 1 year at baseline until they reached age 10 or 11, defined by body mass index (BMI) ≥ 85th percentile, using the Centre for Disease Control and Prevention growth charts. Age at introduction to formula or solids was dichotomized at four months. We used mixed effects logistic regression for performing all analyses with and without adjusting for mother’s BMI, age during pregnancy, and social disadvantage index. Missing data were estimated using multivariate normal imputation having 25 imputations. The odds of overweight or obesity were significantly higher among infants introduced to formula or solids at ≤4 months compared to those introduced at >4 months in both unadjusted (odds ratio = 2.3262, *p* = 0.023) and adjusted (odds ratio = 1.9543, *p* = 0.0475) analyses. The odds of overweight or obesity when age at formula or solids introduction was held fixed at ≤4 months, increased significantly (odds ratio = 2.0856, *p* = 0.0215) for children stopping breastfeeding at age ≤4 months compared to >4 months. Thus, increasing the prevalence of breast-feeding without any formula or solids to 4–6 months in southwest Sydney should be a worthwhile public health measure.

## 1. Introduction

A public health priority has been set in many developed countries for preventing the onset of overweight and obesity amongst children. The most identifiable factors contributing to the early onset of childhood obesity include, among others, infant feeding practices such as breastfeeding, feeding of infant formula and solid foods. Childhood obesity is linked to adolescent and adult obesity as obese children have a 78% and 25–50% risk of progressing to obesity in adolescence and adulthood, respectively [1]. Childhood obesity also increases the risks of medical and psychosocial problems during the childhood period [2]. Five longitudinal studies and three systematic reviews of the relationship between breastfeeding and childhood overweight/obesity conclude that exclusive breastfeeding is a protective factor of overweight [3,4,5,6,7] and obesity [6,7,8,9,10], and sometimes there exists a dose response relationship between the two [5,11,12]. A large number of studies have also shown a strong relationship between early introduction of solids and risk of obesity [3,13,14]. A very large cohort study having more than 10,000 children, known as the United Kingdom (UK) millennium cohort study, found that children had a higher risk to be overweight or obese at three and five years of age when solids were introduced to them before four months, compared to after four months [15]. This association is likely to be modified by age at breastfeeding cessation [16,17]. A meta-analysis by Owen et al. [18] has shown that there is significantly lower risk of obesity among infants across the life course when they were breastfed compared to formula fed during infancy.

The World Health Organization (WHO) now recommends that infants are breastfed exclusively for the first six months of life, partially breastfed for up to two years or beyond, and introduced to solid foods only during the second half of the first year [19]. Australia, among many other countries, has adopted these recommendations.

Among the developed nations, Australia has amongst the highest childhood obesity rates with approximately 20% of children at 2–3 years of age being overweight or obese [20]. Within Australia, southwestern Sydney has the highest childhood obesity rate [21], and early introduction to formula or solids is also quite common [22]. A randomized trial conducted here for assessing childhood obesity intervention, known as the Healthy Beginnings Trial [22], found that out of the 96% of mothers initiating breastfeeding, as high as 58% did not continue breastfeeding to six months, and hence did not meet the WHO recommended guidelines for breastfeeding. One in ten children/teenagers, or more than 20,000 children/teenagers between ages 2 and 17, are obese in the southwestern Sydney local health district or SWSLHD [21]. The issue of childhood obesity in southwest Sydney has been a focus of attention for some time now, but lack of routine data collection has prevented the monitoring of its changes over time, and, hence, it is not clear whether the epidemic is growing [21]. Also, there has been no study to examine the link between early introduction to formula or solid foods and the risk of childhood overweight or obesity and whether this association is modified by child’s age at breastfeeding cessation in the first year, or in other words, by breastfeeding duration in the first year. Hence, the aim of this study was to achieve this task in the southwestern Sydney population and to determine how rate of overweight/obesity has changed over time in this region. 

## 2. Materials and Methods

### 2.1. Study Population

We included 346 infants who at baseline were singleton and full term births and were of normal weight in southwestern Sydney using the Growing Up in Australia: longitudinal study for Australian children (LSAC), which commenced in 2003–2004 with a representative sample of Australian children in two cohorts. These were families with 4–5-year-old children and families with 0 or 1-year-old children. In this study, we used the cohort of families with 0 or 1-year-old children. Data for these children were collected every two years from study informants (until the children turned 10 or 11 years of age) including parents, carers, and teachers when the child was small and from the child when he/she was of an appropriate age. The final round of data collection (wave 10) was completed in late 2013. 

The LSAC investigates the effect of children’s social, economic and cultural environments on their wellbeing over the life course. It has a broad multi-disciplinary base and examines policy-relevant questions about development and wellbeing. The research questions span parenting, family relationships, education, child care, and health. By tracking children (who are now adolescents and young adults) over time, the study can help to understand the factors associated with different developmental pathways. A major aim of the project was to identify policy opportunities for improving support for children, young people, and their families, and to identify opportunities for early intervention. Details of this study’s methodology are given elsewhere [23].

### 2.2. Variables

Child’s weight was measured, according to the data users guide [24], by weighing the child on a Salter Australia glass bathroom scale. It was obtained by weighing the mother or another adult with the infant, then weighing the mother or adult on their own and subtracting the difference. Child’s height was obtained by an Invicta stadiometer (Invicta, London, UK) taking two measurements and averaging them. If the two measurements differed by more than 0.5 cm, a third measurement was taken, and the closest two were averaged. Body mass index (BMI) for age and sex was then calculated for 2/3, 4/5, 6/7, 8/9 and 10/11 year-olds in kg/m^2^ using the directly assessed child’s height and weight. Using the centres for disease control and prevention growth charts [25], BMI *z*-scores were then calculated for these ages of children, and childhood overweight or obesity was defined according to the age- and sex-specific 85th or higher percentiles. Information on infant feeding for the study cohort was retrospectively collected in wave 0 from the mothers when the child was 0/1 year-old. A cut-off point of four months was used to dichotomize age at introduction to formula or solids, as our baseline sample was collected in 2003 prior to the 2004 WHO guideline of six months for introducing supplements or complements. Age at breastfeeding cessation was dichotomised similarly.

### 2.3. Statistical Analysis

Statistical analyses included descriptive analysis presenting several graphical plots and multivariate analysis using mixed effects logistic regression, which accounted for correlated errors associated with repeated, binary, and correlated observations. The covariance structure used was unstructured. The response variable proportions overweight/obese at ages 2/3, 4/5, 6/7, 8/9 and 10/11 years and some of the covariates such as maternal age, BMI during early pregnancy, and social advantage index had missing data, while the infant feeding variables were complete. To adjust for missing data, a missingness model was defined using sociodemographic factors such as maternal age, parity, BMI, education, country of birth, marital status, smoking during pregnancy, and child characteristics like gender; those characteristics that predicted continued participation. Missing data for all analyses were estimated using multivariate normal imputation having 25 imputations. The unadjusted mixed effects logistic regression model (Model 1) considered childhood overweight or obesity as the response variable and follow-up time points and age at introduction to formula or solids as categorical independent variables. The corresponding confounder-adjusted model (Model 2) included the same covariates but in addition included controls for children’s age at breastfeeding cessation (defined as categorical with categories ≤4 months and >4 months), mother’s BMI, age during pregnancy, and social disadvantage index, which were included as continuous covariates. Another model was fitted having the same covariates plus an interaction between age at introduction to formula or solids and age at breastfeeding cessation (Model 3). Finally, a model (Model 4) similar to Model 2 was fitted by including an interaction between age at introduction to formula or solids and another categorical variable accounting for baseline and follow-up time points. Confounders maternal BMI during early pregnancy and social disadvantage index were found to be significant predictors in Models 2, 3 and 4 (results not shown). We used SAS version 9.3 (SAS Institute, Cary, NC, USA) for performing all analyses. For estimating the likelihood ratio, adjusted for using multiply imputed data based on two nested models, we used its mixed effect model version called D3 statistic by Meng and Rubin [26], which follows an approximate F distribution, by adapting the SAS macro written by Mistler [27]. This test was required for assessing the significant effect of an interaction between timing of formula or solids introduction and time when Model 4 and Model 2 were compared.

### 2.4. Demographics of Southwestern Sydney

Southwestern Sydney is the southern part of the Greater Western Sydney which is a large region of Sydney, New South Wales, Australia, covering 5800 km^2^ with an estimated population of 2,232,661 residents in 2016 [28]. Southwestern Sydney consists of 12 local government areas having a population of 820,000 residents. Despite encompassing residents from a range of socioeconomic backgrounds, southwestern Sydney [21] as well as the Greater Western Sydney experience greater disadvantage compared to the rest of metropolitan Sydney, which predisposes its residents to poorer health outcomes [28]. Key sources of health inequality in this population include low income, lack of access to health services, high rates of socioeconomic and ethnic diversity, prevalence of obesity, and fast population growth [28,29]. It is believed that the obesity epidemic could be the driving factor in the poor overall health for southwest Sydney [21]. Within SWSLHD, Canterbury has the highest childhood obesity or overweight rate among all local government areas with 28 per cent of children being obese or overweight followed by Bankstown with 27 per cent of children being overweight or obese [21].

## 3. Results

### 3.1. Descriptive Analysis

Children who were neither born as preterm nor post-term nor were overweight or obese at birth were weighed at age one year and their mean weight was 9.23 kg with a standard deviation (SD) of 1.47. At age 2 or 3 years, child weight increased to 15.05 kg (SD = 2.02), and at ages four, six, eight, and ten, child weight further increased to 19.23 kg (SD = 4.09), 24.85 kg (SD = 4.59), 32.01 kg (SD = 6.83) and 40.51 kg (SD = 9.59), respectively.

**Figure 1:**
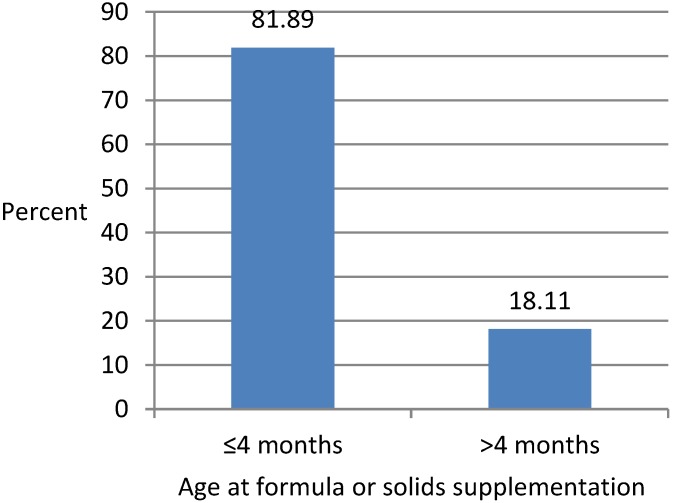
Distribution of age at introduction to formula or solid foods.

The mean age at introduction to formula or solids was 67.79 days (SD = 3.52) or about two months. Figure 1 shows bar chart representation of the distribution of age at introduction to formula or solids. It shows that a vast majority (81.89%) of mothers in southwestern Sydney introduced their infants to formula or solid foods within four months of their birth while the remaining 18.11% of mothers introduced these foods to their infants after four months. Hence, early introduction of supplementary (formula) or complementary (solid) foods was quite common in southwestern Sydney.

**Figure 2:**
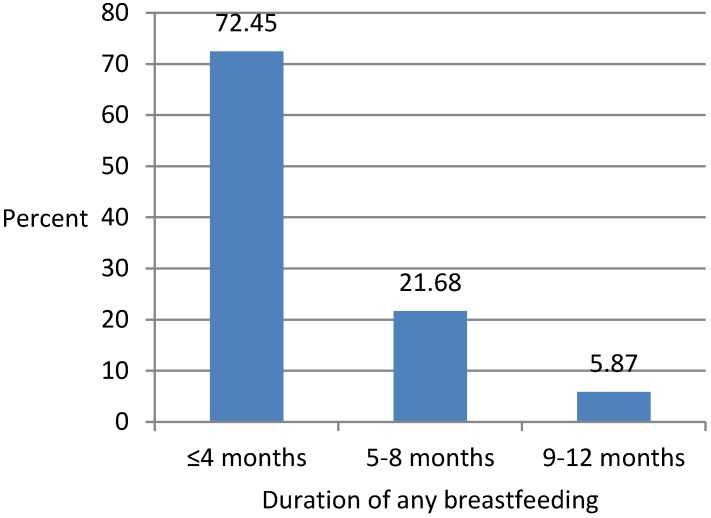
Distribution of age at breastfeeding cessation during first year.

The mean age at stopping of breastfeeding was 92.57 days (SD = 4.91) or about three months. Figure 2 shows a bar chart representation of the distribution of age at stopping of breastfeeding during the first year. Around three-fourths (72.45%) of mothers stopped breastfeeding their infants within four months, around one-quarter (21.68%) stopped breastfeeding their infants at 5–8 months, and the remaining 5.87% stopped breastfeeding their infants at 9–12 months. So, there was no mother breastfeeding her infant for more than a year. 

**Figure 3:**
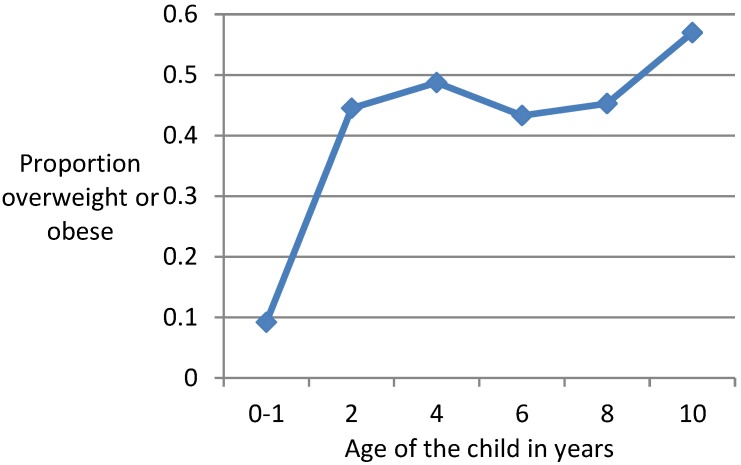
Plot of proportion of overweight or obese children against age of the children (in years).

Figure 3 shows a line graph for proportions of mothers whose children are overweight/obese by ages 2/3, 4/5, 6/7, 8/9 and 10/11 years. Only around one-tenth (9.18%) of the study cohort of mothers either had overweight or obese children at age 0/1 year, which increased five-fold to 44.5% when their children reached age 2/3 years, then there was a slight increase to 48.74% when their children reached age 4/5 but a decline to 43.29% at age 6/7, after which there was a slight increase at age 8/9 (45.3%) but a rapid increase at age 10/11 (57%). This trend suggests that there was an obesity epidemic amongst children of southwestern Sydney with a very rapid increase at age two compared to ages one, however, afterwards there was little change in overweight/obesity rates at ages four, six, and eight, which was followed by a rapid increase at age ten when the overweight/obesity rate was the highest.

### 3.2. Multivariate Analysis

Results of all four models are summarised in Table 1. The unadjusted analysis in Model 1 shows that mothers whose infants were introduced to formula or solids within the age of four months were significantly (*p* < 0.05) and about 2.33 times more likely to become overweight or obese at ages 2/3, 4/5, 6/7, 8/9, and 10/11 years compared with mothers whose infants were introduced to formula or solids after four months. The 95% confidence interval for relative odds also supports the result of the statistical test that age at introduction to formula or solids has significant effect on odds of overweight or obesity. The magnitude of this effect in the adjusted analysis performed in Model 2 is slightly smaller as mothers whose infants were introduced to formula or solids within the age of four months were only about 1.95 times more likely to become overweight or obese compared with mothers whose infants were introduced to formula or solids after four months. The effect is still significant at the 5% level based on t test and 95% confidence interval. 

The results for Model 3 demonstrate that the effect of age at introduction to formula or solids was modified by age at stopping breastfeeding during the first year. The odds of overweight or obesity, when age at formula or solids introduction was held fixed at ≤4 months, increased significantly (*p* < 0.05) with about 2.09 times more children stopping breastfeeding at age ≤4 months compared to those stopping at >4 months.

In the adjusted analysis performed in Model 4 that has an interaction between age at introduction to formula or solids and time waves examined, age at formula or solids introduction does not show any consistently increasing or decreasing effect by time. The effect is not significant at 5% level as confirmed by the non-significant *p*-value for the likelihood ratio test based on multiply imputed data (*p* = 0.4537 for D3 statistic = 0.940773, with degrees of freedom 5 and 830), when Model 4 is compared to the nested model without the interaction term (Model 2). This implies that the effect of age at introduction of formula or solids on the odds of childhood overweight or obesity is time invariant. 

## 4. Discussion

The Australian region of southwestern Sydney is characterised by early introduction of supplementary and complementary foods as well as a high prevalence of childhood overweight or obesity. This region is also socioeconomically diverse and disadvantaged. We found that in southwestern Sydney, there was a five-fold increase in the overweight or obesity rate to 44.5% at the second or third year of life for children aged 0 or 1 year at baseline, which remained almost stable at the fourth or fifth, sixth or seventh, and eighth or ninth year of life for these children while increasing 12% at the tenth or eleventh year of life compared with that at the eighth or ninth year of life. In other Australian populations such as in western Australia, similarly high overweight or obesity rates have been observed after the first year of life of children, although the trends seem to differ from this study [4,30]. The high rates for childhood overweight or obesity are likely to be linked to increasing overweight or obesity rates among adults, supporting the projection of increasing overweight or obesity rates amongst adults in Australia [31].

In our study cohort, about 82% of mothers introduced formula or solids to their children within the first four months of their lives, indicating that early introduction to these foods was very common in the region. About 94% of mothers stopped breastfeeding within eight months of the birth of their children. This study demonstrated the increased risk of childhood overweight or obesity associated with early introduction of formula or solids in this region using mixed effects logistic regression. Due to the use of a longitudinal study design and fulfilment of the key criteria for causality [32], this association may be interpreted in terms of a causal relationship, as such, early introduction of formula or solids may be considered as a risk factor of childhood overweight or obesity in the region. However, the children were not randomized into exposure and non-exposure groups at baseline to control for potential confounding bias, so we need to be cautious about interpreting the results causally. The increased risk of childhood obesity by early formula or solids feeding had been speculated previously, but lack of any longitudinal study to examine this association prior to use of LSAC in this study prevented us knowing whether the association holds longitudinally. This motivated us to conduct this study, which was facilitated by the availability of a cohort of mothers in the southwestern Sydney population, from the nationally representative LSAC dataset. 

The primary exposure variable of interest, age at introduction to formula or solids, was dichotomized using a four-month cut-off point, as our baseline sample was collected in 2003 just prior to the 2004 WHO guideline of six months for the introduction of supplementary or complementary foods to infants. This approach is justifiable as prior to 2004 the WHO guideline for introducing these foods was four months. Our results support other studies which also found that children introduced to formula within four months have increased risk of overweight in western Australia [4], obesity in the United States (US) [16,33] and overweight or obesity in the UK based on a very large cohort [15]. It must be mentioned that the type of infant/follow-up formulas in Australia in the year 2003 differed substantially from the ones that are used today, in particular, the quantity of protein is much lower and protein quality has improved. The aim of the study was not to examine the impact of quantity and quality of protein intake by infants on childhood obesity risks. In our study cohort, there were only a small number of mothers who introduced formula or solids to their infants above the age of six months, so there was inadequate statistical power to estimate the effect of age at introduction to formula or solids when it was dichotomised by a six-month cut-off point. A larger cohort of mothers might help to perform such analysis which could be the scope of a future study. We further found that the effect of age at introduction to formula or solids was modified by age at breastfeeding cessation during the first year with a two-fold increase in odds of overweight or obesity among those stopping breastfeeding within four months and introducing formula or solids within four months. A similar effect of modification was found in another study based on the US population when the outcome was odds of obesity alone [16], although the effect size was much higher at around six-fold.

This study has several strengths and a few limitations. An important strength of this study is its longitudinal study design. Many similar previous studies were restricted to cross-sectional analyses and, therefore, any association found in those studies cannot be interpreted temporally. We excluded from our study cohort mothers who at baseline had multiple and pre term births and whose babies were not of normal weight. We were able to adjust or control for the key confounders in our multivariate analysis while assessing our exposure-outcome relationship. While there were only three confounders adjusted, maternal age, BMI during early pregnancy as a proxy for the confounding variable pre-pregnancy BMI, and maternal social advantage index, we also explored for other potential confounders like maternal education, country of birth, marital status, parity, smoking during pregnancy, and child characteristics like gender, however, these were not found to be confounders while the three we adjusted for were found to be confounders. Thus, while adjustment for confounding using the three variables reduced the magnitude of excess risk associated with early introduction to formula or solids, we considered that further adjustment for residual confounding was unlikely to remove the adjusted effect estimated in this study. It may be mentioned that maternal pre-pregnancy BMI has been suggested to be a confounder in infant feeding-childhood obesity association studies [4,30,34]. In LSAC, maternal BMI during pregnancy was measured during early first semester of women. Maternal BMI during pregnancy, particularly at an early first trimester, has been found to be a good proxy for pre-pregnancy BMI of mothers as there is minimal change between pre-pregnancy BMI and BMI measured at early first trimester [35]. Also, for pre-pregnancy weight, as high as 91%–95% women are found to be correctly classified based on a first trimester weight [36] and hence maternal BMI measured at first trimester is a good proxy of pre-pregnancy BMI. Due to not assessing information on infant feeding factors regularly from a mother throughout her child’s infancy, the recall period for obtaining information on infant feeding factors was kept to within one year since the child was not over one-year-old when the mother was asked about these practices. This reduced possibilities of recall bias. Children’s BMI measures were reasonably accurate as children’s weight and height were both obtained by anthropometric methods rather than self-reports. In particular, children’s weights were measured using the Salter Australia glass bathroom scale and heights were obtained by an Invicta stadiometer taking two measurements and averaging them. If the two measurements differed more than 0.5 cm, a third measurement was taken and the closest two were averaged. In our survey questionnaire, a question was asked to mothers for the age at which their children were either introduced to formula or solids, without distinguishing which non-breastmilk food was given first and at what age. This did not allow us to estimate the risk of childhood overweight or obesity associated with age at solids introduction, separately for breastfed and formula-fed children, although some studies have found these risks to differ [35]. For a cohort of 346 children at baseline, 49 (14.16%) dropped out at age 2/3, 63 (18.21%) dropped out at age 4/5, 80 (23.12%) dropped out at age 6/7, 99 (28.61%) dropped out at age 8/9, and 140 (40.46%) dropped out at age 10/11. These missing data were estimated by multiple imputation which is a well-established method for estimating missing data. This study did not examine what kind of food was introduced, for example, the impact of vegetables is not the same as cereals with plenty of sugar. This is a limitation of the study. Despite having a moderate but not large cohort size, we were still able to find a strong association in this study between our dichotomous exposure and outcome variables after controlling for the key confounders. A range of sample size calculations prior to commencing the analysis using a typical 5% type I error and 80% power revealed that our existing cohort size met the minimum expected sample size required to detect the clinically significant effect size (based on expert opinion) for our exposure-outcome relationship. So our analysis may provide a conservative estimate of *p*-value for the effect of our exposure variable as well as its effect size (odds ratio) for assessing the exposure-outcome relationship, and hence using a larger cohort may result in a stronger association in terms of *p*-value and/or effect size. To provide a balance between the descriptive use of sample effect size and statistical significance, we presented confidence intervals of estimated effect size (odds ratio). This approach is very useful for translating research findings to education practice as it not only allows for the assessment of whether a sample based result is likely, but also if an effect is practically noteworthy and replicable [37]. Although the dose-response relationship between breastfeeding duration and risk of childhood overweight or obesity has not been studied in southwestern Sydney, this can be considered as a topic for a future study given that in another Australian population in south Australia, a dose-response relationship between the two variables has recently been found for two and three-year-old children during follow-up [38]. 

## 5. Conclusions

We have showed in this study that in terms of infant feeding patterns, the first four months of life in infants is the time period of greatest risk for development of obesity later in childhood. We recommend continued exclusive breastfeeding for 4–6 months and not over six months as it may result in mothers exclusively breastfeeding, for example, for 9 months which is not recommended. There is a two-fold increase in odds of overweight or obesity among those breastfed (any form) within four months and introduced to formula or solids within four months, compared to those breastfed (any form) longer and introduced to formula or solids later. To reduce childhood obesity risk associated with early supplementation or complementation of infants, SWSLHD has been proactive in partnering with pilot programs to complement other programs offered at child care through to high school students. This SWSLHD pilot program, known as the Chat Study, provides mothers who give birth at Liverpool and Campbelltown hospitals in southwest Sydney with a regular text message or phone call offering nutritional coaching to help with healthy development, including discouraging early introduction of supplementary and complementary foods. SWSLHD has strongly suggested to the local government and other stakeholders in the past few years that they should increase the region’s green space and water refill stations and put more gyms in parks. These facilities are growing fast and, therefore, are expected to further facilitate physical and sporting activities of children. Residential and parkland areas are fast growing in this region too. Considering that these contribute to the majority of green space, this is encouraging. With reductions in modifiable childhood obesity risk factors such as early introduction to formula or solids and physical inactivity [39], among others, it is expected that childhood overweight or obesity rates will decline in future. However, it may still take several decades for these rates to get back to normal as past experience with regards to the effect of smoking rate reductions may indicate [21].

## Figures and Tables

**Table 1:** Estimated odds ratios, 95% confidence intervals (CI) and *t* statistic for the effects of age at introduction to formula or solids, the interaction effects between this variable and age at breastfeeding cessation, and timing of follow-up waves on odds of overweight or obesity.

Covariate	Odds Ratio	95% CI	*t* Ratio
**Model 1** Age at starting formula or solids ≤4 months vs. >4 months	2.3262	1.8025–2.4132	2.3026 ^a^
**Model 2** Age at starting formula or solids ≤4 months vs. >4 months	1.9543	1.1010–3.4687	2.27 ^a^
**Model 3** Age at starting formula or solids * age at breastfeeding cessation ≤4 months * ≤4months vs. >4months * >4 months	2.0856	1.1225–4.8205	1.9845 ^a^
**Model 4** Time*Age at starting formula or solids			
Age 2/3 * ≤4 months	0.3569	0.0668–1.9058	−1.21
Age 4/5 * ≤4 months	0.8437	0.1547–4.6014	−0.20
Age 6/7 * ≤4 months	0.9050	0.1564–5.2385	−0.11
Age 8/9 * ≤4 months	0.4573	0.0810–2.5828	−0.89
Age 10/11 * ≤4 months vs. Age 0/1 * >4 months	0.6093	0.1001–3.7088	−0.54

Notes: * indicates product sign. All models controlled for the effect of time while models 2–4 also controlled for children’s age at breastfeeding cessation, maternal age, body mass index (BMI) during pregnancy, and social advantage index; the statistical tests are performed at 5% level of significance; the superscript ^a^ indicates *p* < 0.05.
